# Enhancing Formaldehyde Selectivity of SnO_2_ Gas Sensors with the ZSM-5 Modified Layers

**DOI:** 10.3390/s21123947

**Published:** 2021-06-08

**Authors:** Wei Wang, Qinyi Zhang, Ruonan Lv, Dong Wu, Shunping Zhang

**Affiliations:** 1 School of Materials Science and Engineering, Wuhan University of Technology, Wuhan 430070, China; ww18571471955@whut.edu.cn (W.W.); lrnrita0228@163.com (R.L.); dongwu@whut.edu.cn (D.W.); 2 School of Materials Science and Engineering, Huazhong University of Science and Technology, Wuhan 430074, China; pszhang@mail.hust.edu.cn

**Keywords:** ZSM-5 zeolite, SnO_2_ gas sensor, formaldehyde, selectivity

## Abstract

High performance formaldehyde gas sensors are widely needed for indoor air quality monitoring. A modified layer of zeolite on the surface of metal oxide semiconductors results in selectivity improvement to formaldehyde as gas sensors. However, there is insufficient knowledge on how the thickness of the zeolite layer affects the gas sensing properties. In this paper, ZSM-5 zeolite films were coated on the surface of the SnO_2_ gas sensors by the screen printing method. The thickness of ZSM-5 zeolite films was controlled by adjusting the numbers of screen printing layers. The influence of ZSM-5 film thickness on the performance of ZSM-5/SnO_2_ gas sensors was studied. The results showed that the ZSM-5/SnO_2_ gas sensors with a thickness of 19.5 μm greatly improved the selectivity to formaldehyde, and reduced the response to ethanol, acetone and benzene at 350 °C. The mechanism of the selectivity improvement to formaldehyde of the sensors was discussed.

## 1. Introduction

Formaldehyde is a kind of toxic gas that is harmful to human health. Occasionally formaldehyde inhalation up to a certain amount usually causes headache and limb fatigue, and a long term severe inhalation of formaldehyde ultimately damages the human being’s kidneys and other organs, even destroys the brain nerve system [1,2]. The research on rapid detection of formaldehyde can contribute to early prevention and treatment of its pollution and therefore beneficial to the healthcare of the person who may contact this harmful substance by chance. Recently gas sensors are playing an important role in detection of formaldehyde [3,4,5]. At present, a number of metal oxide semiconductors (MOSs) for formaldehyde measurements have been reported, such as NiO [6], ZnO [7], SnO_2_ [8], WO_3_ [9], In_2_O_3_ [10], Co_3_O_4_ [11], and so on. Among the semiconductor materials, SnO_2_ is most widely used to measure volatile organic compounds (VOCs) gas in gas sensors. However, SnO_2_ gas sensors are easily interfered by other gases in their applications, so the sensitivity and selectivity need to be strengthened and improved [12]. It was showed that the performance of the SnO_2_ gas sensors could be optimized by controlling the grain size of SnO_2_ [13], doping [14], and surface modification [15]. Surface modification is also an appropriate means to enhance selectivity.

Zeolites show movable frame and sieve pore structure, which is widely utilized in catalytic and plasma exchange applications [16,17]. Considering zeolite can act as a molecular sieve due to its structure with tiny pores, the selectivity of the sensors are greatly improved when zeolite is coated on the sensitive material as a modified layer [18,19,20,21,22]. Vilaseca et al. coat the Pd/SnO_2_ surface with a non-continuous zeolite A (LTA) layer by microdropping from a zeolite suspension. The hydrocarbon (methane and propane)/ethanol mixtures were measured and the sensors presented remarkable filtering capacity [18]. ZSM-5 and silicalite-1 are the most commonly applied zeolites to improve the selectivity of gas sensors [19,20,21,22]. The [010] highly preferred-orientation silicalite-1 layer coated on SnO_2_ thin film sensor was proved to be able to resist the influence of H_2_O when detecting C_2_H_4_ [19]. In particular, the selectivity of metal oxide semiconductor (MOS) gas sensors is very poor [19]. zeolites are very effective for improving the selectivity of MOS gas sensors to formaldehyde, whether they are used as external [20] or in-situ membranes [21,22]. When an external 3 μm zeolite MFI film is placed in front of the gas sensor, it is difficult for VOCs, such as acetone, ethanol, and isoprene, to pass through the film and react with the sensitive material except formaldehyde [20]. Sun et al. modified the SnO_2_ sensors with different SiO_2_/Al_2_O_3_ ratios and different particle sizes of ZSM-5 to explore the influence on the gas sensitivity of the SnO_2_ sensor. They found that the response value of the SnO_2_ gas sensor coated with ZSM-5 molecular sieve (SiO_2_/Al_2_O_3_ = 70, particle size 300 nm) to formaldehyde gas increased, meanwhile the response value to acetone decreased, therefore improved the selectivity [21]. Sun et al. also proved that the SnO_2_/ZSM-5 composite nanofibers could resist the interference of ethanol and acetone when formaldehyde was measured [22].

Although many studies have shown that zeolite membrane improves the selectivity of gas sensors to formaldehyde, the mechanism of the selectivity enhancement is still unclear. It is well known that zeolite membrane plays the role of filtration and catalysis when it interacts with gas molecules. The thickness of the zeolite membrane is an important factor affecting the selectivity of gas sensors besides the composition, the grain size [21], and the morphology [22] of the zeolite membrane, because the membrane thickness affects the diffusion of the gas molecules, which is crucial to both catalysis and filtration. However, there are few reports discussing the influence of zeolite thickness on the gas sensitivity of MOS sensors to formaldehyde. In this paper, ZSM-5 was chosen as the modified layer to enhance the formaldehyde selectivity of MOS gas sensors. We developed a novel simple low-cost screen printing technique to prepare zeolite film, utilized this technique to control the thickness of the zeolite modified layer. The SnO_2_ sensors with different thickness of the zeolite modified layer were exposed to several VOCs gases. The selectivity of the sensors to formaldehyde was examined, and the mechanism of the selectivity improvement of the sensors was also discussed.

## 2. Materials and Methods

### 2.1. Preparation of Nano-SnO_2_

Nano-SnO_2_ was prepared by thermal decomposition of tin oxalate salt. Tin sources (dihydrate and tin chloride), hydrochloric acid, and ethanedioic acid dihydrate were purchased from Sinopharm Chemical Reagent Co., Ltd. (Shanghai, China). The typical preparation manner was as follows: First, 4.513 g SnCl_2_·2H_2_O was weighed and dissolved in 30 mL deionized water. The predetermined amount of hydrochloric acid (12 mol/L) was added to adjust the pH value of the solution to 3, and then the solution was heated, boiled, and stirred until the solution became clear. Subsequently, a certain amount of C_2_H_2_O_4_·2H_2_O was added to the solution to form a stannumite oxalate precipitant. The molar ratio of SnCl_2_·2H_2_O and C_2_H_2_O_4_·2H_2_O was 1:1 to obtain the white stannous oxalate precipitation. The precipitant was filtered and washed, dried at 90 °C for 2 h, and calcined at 600 °C for 5 h. Finally, the nano-SnO_2_ were prepared.

The crystal phases of the nano-SnO_2_ powders were characterized via X-ray diffraction analysis (XRD, D8 Adwance, Bruker, Karlsruhe, Germany). Morphology of the nano-SnO_2_ powders was observed by a transmission electron microscope (TEM, JEM2100F STEM/EDS, JEOL, Tokyo, Japan).

### 2.2. Fabrication of Sensors

**Figure 1:**
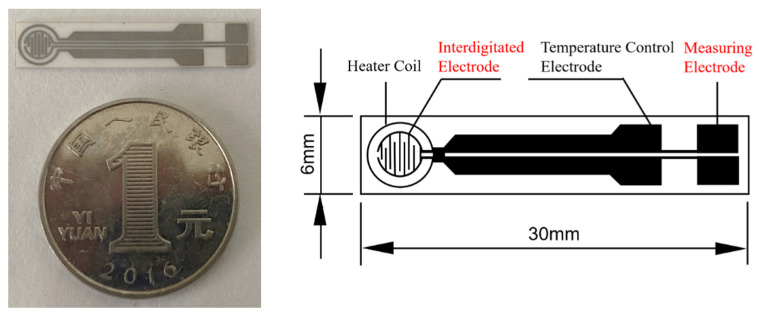
Photo and schematic diagram of the TC-5010 sensor substrate.

TC-5010 sensor substrates (Wuhan Huachuang Ruike Co., Ltd., Wuhan, China) were used in the experiment (Figure 1). The substrates with the size of 30 mm × 6 mm × 0.625 mm is made of Al_2_O_3_ ceramic sheets while the Pt electrode are printed on the substrate. The Pt electrode consists of a heater coil, temperature control electrode, interdigitate electrode, and measuring electrode. The sensitive material is screen printed on the interdigitate electrode. The sensing film is a circle with a diameter of 3.2 mm. The working temperature of the sensing film is automatically controlled by the input power to the heating coil because Pt has a good linear resistance to temperature relationship.

The prepared nano-SnO_2_ powders and printing oil (YY-1010,Wuhan Huachuang Ruike Co., Ltd., Wuhan, China) were mixed and ground into the paste with the mass ratio of 1:1. The printing oil is mainly composed of terpineol, butyl carbitol acetate, dibutyl phthalate, and ethyl cellulose. The paste was screen printed on the interdigitate electrode within 5 min to prevent the printing oil loss due to volatilizing. Then, the printed sensors (named S(C)) were dried at 60 °C for 1 h, and sintered at 600 °C in air for 2 h.

The commercially available ZSM-5 zeolite (Si/Al ratio: 70, XFNANO, Nanjing, China) was purchased and used for preparation of the modified layers. The ZSM-5 zeolite layers were also screen printed on the surface of the S(C) gas sensors. Preliminary exploratory experiments showed that the film thickness after sintering is about 10 μm for the nano-SnO_2_ layer and more than 30 μm for the ZSM-5 zeolite layer, respectively, when the mass ratio of the raw material and the printing oil is 1:1. The ZSM-5 zeolite layer was thicker than the nano-SnO_2_ layer because ZSM-5 zeolite had higher activity and specific surface area. The performance of the sensors with the thick ZSM-5 zeolite modified layer was very poor. Reducing the mass percentage of the ZSM-5 zeolite in the ZSM-5 zeolite and the printing oil paste will help to obtain a thinner modified layer. After repeated experiments, the mass ratio of the ZSM-5 zeolite and the printing oil was finally set to 1:5. The screen printing process of the ZSM-5 modified layer is the same as that of the nano-SnO_2_ film, except that the mass ratio of the ZSM-5 to the printing oil is changed to 1:5. The ZSM-5 modified layers with different thickness were obtained by simply repeating the above printing process. After printing each ZSM-5 modified layer, the printed sensor was dried at 60 °C for 10 min to improve the stability of the as-printed layer. The screen printing process of the ZSM-5 modified layer was repeated 2, 3, 4, 5, and 10 times, named S(C/Z2), S(C/Z3), S(C/Z4), S(C/Z5), and S(C/Z10), respectively. The structure of the gas sensors were listed in Table 1. Finally, all the sensors were dried at 60 °C for 1 h, then sintered in air at 400 °C for 2 h with the heating rate of 5 °C/min. All the sensors were aged at 400 °C for 3 days to improve the stability of the sensors before the measurement.

The crystal phases of the commercial ZSM-5 zeolite were characterized via X-ray diffraction analysis (XRD, D8 Adwance, Bruker, Karlsruhe, Germany). Morphology of the commercial ZSM-5 zeolite was observed by a scanning electron microscope (SEM, Zeiss Utral Plus, Cari Zeiss AG, Jena, Germany) and a transmission electron microscope (TEM, JEM2100F STEM/EDS, JEOL, Tokyo, Japan). The specific surface area and pore size distribution were measured by the Brunauer–Emmett–Teller (BET) method using a N_2_ adsorption isotherm (BET, TriStar Ⅱ 3020, Micromeritic, Norcross, GA, USA).

The surface and the cross-sections morphology of the sensors were observed by a scanning electron microscope (SEM, Zeiss Utral Plus, Cari Zeiss AG, Jena, Germany). The element distribution of the product was analyzed by energy dispersive spectroscopy (EDS, JXA-8230, Tokyo, Japan).

### 2.3. Measurement of Sensing Performance

**Figure 2:**
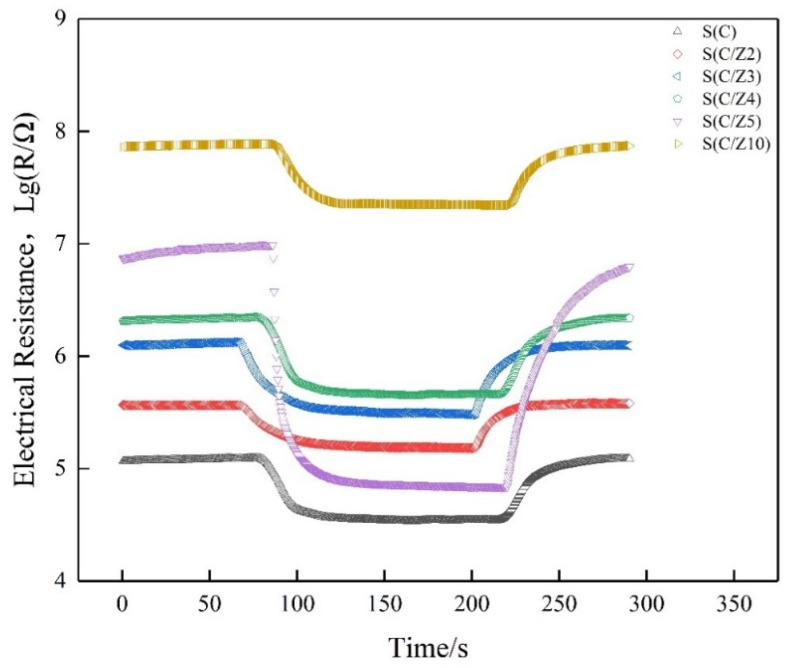
Response transients of the sensors to 100 ppm formaldehyde at 350 °C.

Gas-sensing performance was measured by a commercial SD-101 gas sensing performance testing device (Wuhan Huachuang Ruike Tech. Co. Ltd., Wuhan, China), which can be simultaneously used to test four gas sensors. Volatile gases such as ethanol, acetone, benzene, and formaldehyde were measured by a static method at the concentration of 100 ppm (*v*/*v*). The sensors worked at the temperature of 200 °C, 250 °C, 300 °C, 350 °C, and 400 °C, respectively. During the entire tests, the ambient temperature is 18–20 °C and the relative humidity is 75%–80%. Taking formaldehyde as an example, a typical test procedure was as follows: (1) the sensors were mounted on the SD-101 device and exposed to air in a chamber with 50 L in volume for 5 min at a constant operating temperature while the resistances of the gas sensors stabilized in air; (2) the corresponding quantity of formaldehyde was injected by a microinjector on a heating panel in the chamber, the vaporized formaldehyde vapor was dispersed throughout the air in the chamber by two fans; (3) the resistances of the sensors stabilized again after the fans were shut down to keep the air static in the chamber; (4) the chamber was opened and fresh air was fed into the chamber, the sensors recovered before next measurement. The response transients of the sensors were shown in Figure 2. The responses of the sensors both in air and in testing gas are stable. The typical response and recovery times of the sensors are 35 s and 60 s, respectively. More details about the testing procedure could be found in References [23,24].

The response (S) of the sensors is defined as the ratio of the resistances of the sensors in air (R_air_) to the resistances in the test gases (R_gas_), respectively. In order to compare the selectivity of the sensors, the selectivity coefficient (D) is defined as the ratio of the responses of the sensors with the modified ZSM-5 layers to those of the sensor without the modified layer (i.e., the S(C) sensor) under the same conditions (S_x_/S_0_).

## 3. Results and Discussion

### 3.1. Characterization of Nano-SnO_2_ and ZSM-5

**Figure 3:**
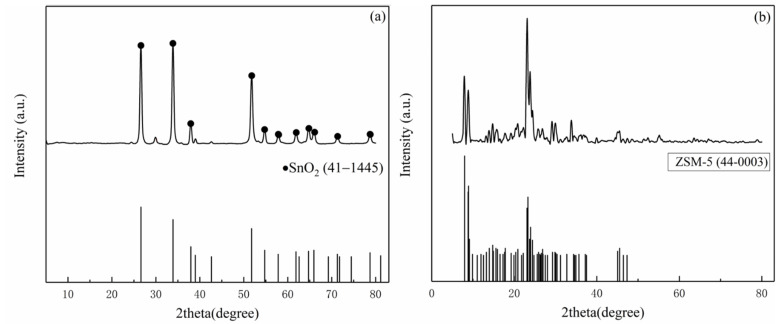
XRD patterns of the samples: (**a**) the nano-SnO_2_ and (**b**) the commercial ZSM-5.

Figure 3 shows the XRD patterns of the nano-SnO_2_ and the commercial ZSM-5. The prepared nano-SnO_2_ powders had peaks in accordance with PDF card No. 41-1445. It is proved that the nano-SnO_2_ powders were successfully prepared by thermal decomposition of stannous oxalate and had a typical tetragonal rutile structure (Figure 3a). The XRD pattern of the commercial ZSM-5 zeolite shows two sharp peaks near 2θ = 7° and 8°, and three strong peaks near 2θ = 23–25°.The position of peaks was consistent with that of PDF card “PDF 43-0003” (Figure 3b).

**Figure 4:**
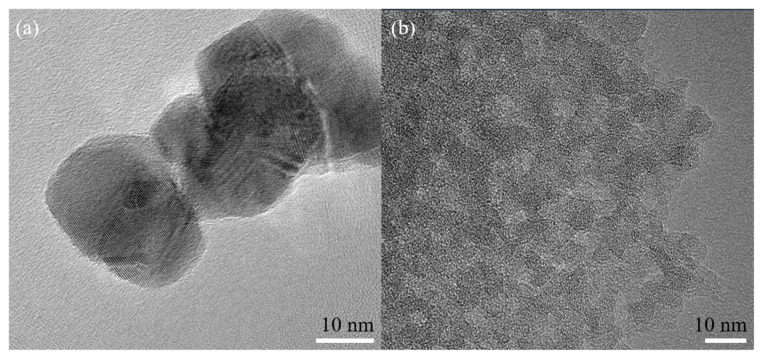
TEM images of the samples: (**a**) the nano-SnO_2_ and (**b**) the commercial ZSM-5.

**Figure 5:**
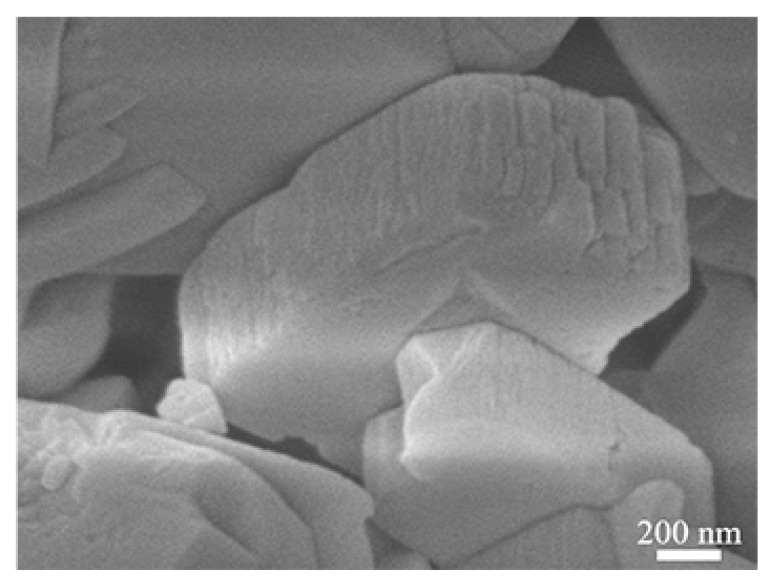
SEM images of the commercial ZSM-5.

Figure 4 shows the TEM images of the nano-SnO_2_ powders and the commercial ZSM-5 zeolite. It can be seen that the nano-SnO_2_ particles show an irregular spherical shape and the size of the nano-SnO_2_ particle was about 40 nm (Figure 4a). Correspondingly, the ZSM-5 zeolite particles were prismatic, and the length of the prisms was about 900 nm (Figure 5). It was revealed that there are plenty of ordered tiny pores, which their size is about 2 nm, in the ZSM-5 zeolite particle as observed in Figure 4b.

**Figure 6:**
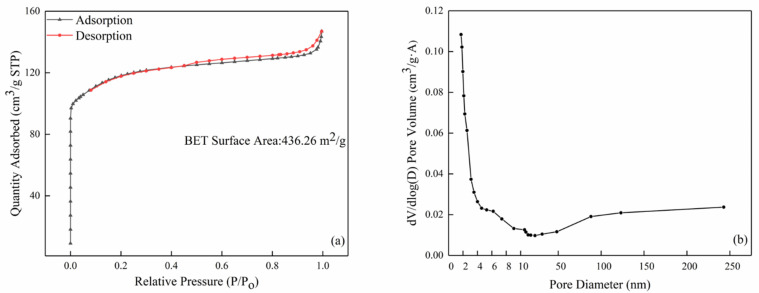
The BET results of the commercial ZSM-5: (**a**) nitrogen adsorption and desorption isotherm curve and (**b**) pore size distribution.

The nitrogen adsorption/desorption isotherm and pore size distribution of the commercial ZSM-5 zeolite are shown in Figure 6. It is clear that the ZSM-5 zeolite shows a large specific surface area of 436.26 m^2^/g and the average pore size was 2.039 nm. The pores in the ZSM-5 zeolite were mainly distributed between 1.9 and 2 nm according to the pore size distribution curve (Figure 6b), which was in good agreement with the TEM result.

### 3.2. Micromorphology Characterization of Gas Sensors

**Figure 7:**
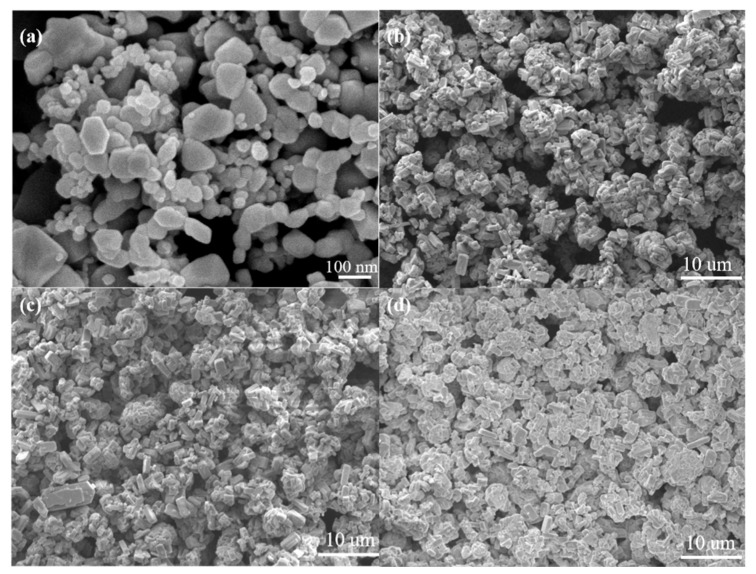
SEM micrographs of surface morphology of the sensors: (**a**) S(C), (**b**) S(C/Z3), (**c**) S(C/Z5), and (**d**) S(C/Z10).

Figure 7 demonstrates the SEM micrographs of the surfaces of the sensors with and without the modified ZSM-5 layer. As shown in Figure 7a, the nano-SnO_2_ particles maintain the original shape and size, and sintering necks between the particles appears after sintering. Several nano-SnO_2_ particles gather together and grow into a larger particle with a diameter of about 100 nm in some places. In addition, the nano-SnO_2_ film is non-continuous. There are many sintered macropores with a size of several microns in the nano-SnO_2_ sensitive film. These sintered macropores are left by volatilizing the printing oil on the sintering process, which are helpful to improve the specific surface area of the sensitive film, then the sensitivity of the sensor. The particle size in the ZSM-5 modified layer was obviously larger than that in the nano-SnO_2_ film. Most of the ZSM-5 prisms aggregated to form secondary particles with the size of about 10 microns (Figure 7b–d). With the increase of the screen printing times of the ZSM-5 layer, the number and size of sintered macropores decreased greatly, and the ZSM-5 layers became more and more compact. When the ZSM-5 layer was screen printed for 3 times, the nano-SnO_2_ particles could not be seen on the surface, and the size of the sintered macropores was about 10 microns (Figure 7b). When the screen printing times increased to 5, most of the sintered macropores reduced to 2–3 microns in size (Figure 7c). The S(C/Z10) sensor had the same size of the sintered macropores as that in the S(C/Z5) sensor, but the number of the sintered macropores was much less (Figure 7d).

**Figure 8:**
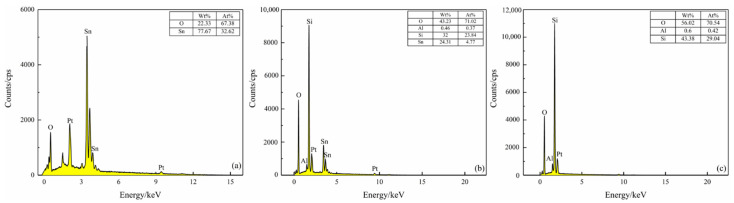
EDS spectrum of sensors surface: (**a**) S(C), (**b**) S(C/Z3), and (**c**) S(C/Z5).

Figure 8 shows EDS analysis of the surfaces of the S(C), S(C/Z3), and S(C/Z5) sensor. The peaks of Pt in EDS spectrum come from the Pt electrode. It can be seen that only two elements, O and Sn, were detected on the surface of the S(C) sensor. The atomic ratio of O to Sn is about 2:1, which is consistent with the stoichiometric ratio of SnO_2_ (Figure 8a). After screen printing for 3 times. the S(C/Z3) sensors surface contained Si, Al, and a small amount of Sn elements, indicating that the ZSM-5 zeolite already printed on the surface of the S(C/Z3) sensor (Figure 8b). The existence of the Sn element in the EDS spectrum implied that the nano-SnO_2_ film was not completely covered by the ZSM-5 layer because of the sintered macropores discussed above. Moreover, only three elements, Si, Al, and O, existed on the surface of the S(C/Z5) sensor, and the atomic ratio of Si to Al was close to 70 (Figure 8c). The absence of Sn element on the surface of the S(C/Z5) sensor indicates that the ZSM-5 layer had completely covered the nano-SnO_2_ film.

**Figure 9:**
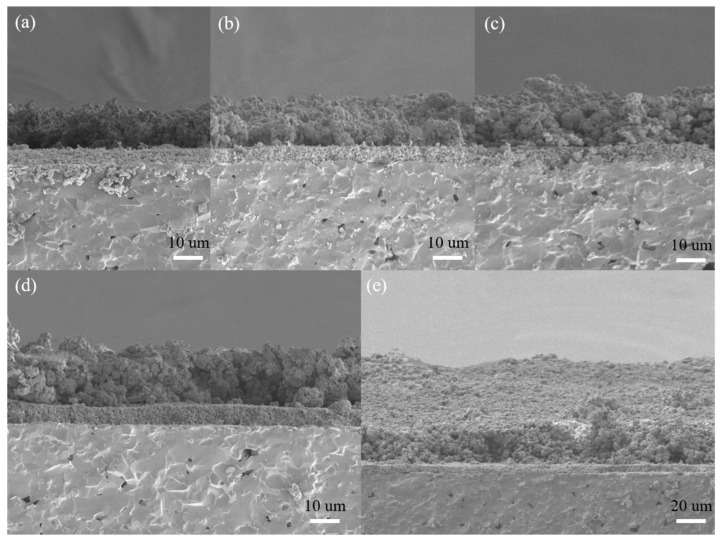
SEM micrographs of the cross-sections of the sensors: (**a**) S (C/Z2), (**b**) S (C/Z3), (**c**) S (C/Z4), (**d**) S (C/Z5), and (**e**) S (C/Z10).

The micrographs of the cross section of the sensors are shown in Figure 9. It is shows that the nano-SnO_2_ film was denser with the thickness of about 5 μm while the ZSM-5 modified layers showed relatively loosened morphology. There was an obvious interface between the ZSM-5 modified layer and the nano-SnO_2_ film. However, fabricated with the same materials and printing manner, the ZSM-5 modified layers had no stratification to each other. The average thicknesses of the ZSM-5 modified layers were listed in Table 2.

It is worth noting that the thickness of the ZSM-5 modified layer was not linear with the number of screen printing process. When the ZSM-5 layer was screen printed twice on the surface of the nano-SnO_2_ film, the thickness of the modified layer reached 12.6 μm. Before increasing the number of screen printing process to 5, the thickness of the modified layer only increased by 1–2 μm after each screen printing. The reason for the above phenomenon is that some organic compounds in the printing oil volatilized, resulting in the decrease of density and increase of porosity of the as-printed film during the drying at 60 °C for 10 min after each screen printing. The next printing paste filled the pores of the as-printed film, so the film thickness increased slowly, but the film density increased rapidly. The surface morphology of the sensors with the ZSM-5 modified layer also confirmed that the S(C/Z5) sensor was much denser than the S(C/Z3) sensor (Figure 7). When the sensors were screen printed with the modified layer five times, the ZSM-5 layer was pressed tight enough, and the thickness of the modified layer increased rapidly with the increase of the number of the screen printing process.

### 3.3. Resistance of Sensor in Air

**Figure 10:**
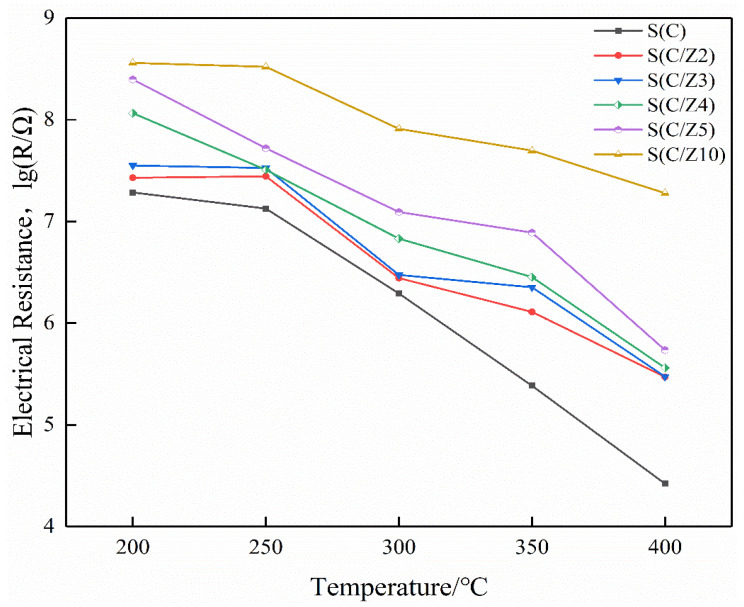
The electrical resistances of the sensors in air.

Figure 10 is the electrical resistances of the sensors working at different temperatures in air. It can be seen that the resistances of all the sensors decreased with the increase of the operating temperature in air. As a semiconductor, the current-carrying electrons in SnO_2_ increased when it was heated, resulting in a decrease in its resistance. It is also shown that the resistances of the modified S(C/Z2), S(C/Z3), S(C/Z4), S(C/Z5), and S(C/Z10) sensors with the modified layer were higher than that of the S(C) sensor without the modified layer in air. The electrical resistances of sensors had a strong correlation to the amount of oxygen absorbed. The more oxygen absorbed, the higher the electrical resistances are. The main components of ZSM-5 were oxides with a movable skeleton structure composed of Si and Al, and the oxygen in the skeleton can diffuse to the surface of the nano-SnO_2_ film. With the increase of the thickness of the modified ZSM-5 layers, more and more oxygen in the skeleton migrate to the surface of the nano-SnO_2_ and became the adsorbed oxygen. The adsorbed oxygen takes electrons away from the nano-SnO_2_ film, leading to the increase in the electrical resistances of the sensors in air.

### 3.4. Gas Sensitive Performance of Sensors

**Figure 11:**
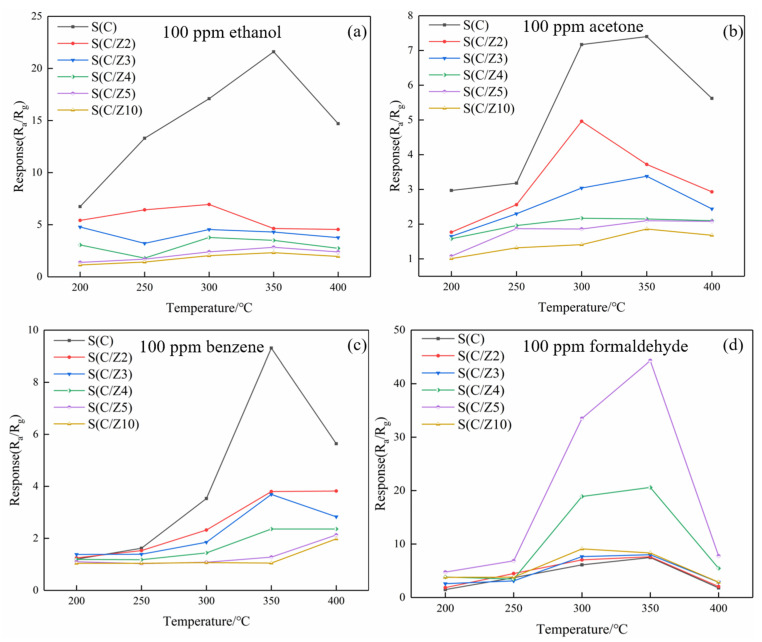
Response values of the sensors to VOCs at different temperatures: (**a**) 100 ppm ethanol, (**b**) 100 ppm acetone, (**c**) 100 ppm benzene, and (**d**) 100 ppm formaldehyde.

Figure 11 shows the response values of the sensors to different testing gases at different temperatures. As can be seen from Figure 11a, the responses of the sensors with the modified layer to ethanol decreased significantly, and the optimal operating temperature of the sensors to ethanol was 350 °C. The variation of the responses of the sensors with the modified layer to acetone and benzene were basically similar to ethanol, and the responses of the sensors to acetone and benzene decreased gradually with the increase of the thickness of the ZSM-5 modified layer (Figure 11b,c). The responses of the sensors with the modified layer to formaldehyde were quite different. The ZSM-5 modified layer greatly improved the responses of the sensors to formaldehyde. When the sensors with the modified layer worked at 350 °C, the response of the S(C) sensor was 7.48–100 ppm formaldehyde. Simultaneously, the responses of the S(C/Z4) and S(C/Z5) sensors were 20.6 and 44.3, respectively, to 100 ppm formaldehyde at the same temperature.

**Figure 12:**
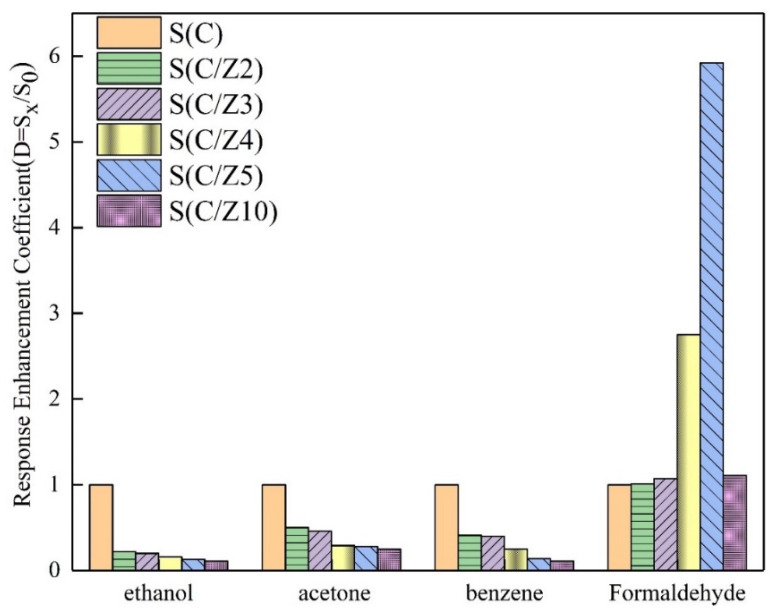
Response enhancement coefficients of the sensors to ethanol, acetone, benzene, and formaldehyde at 350 °C.

In order to further compare the selectivity of the sensors with and without the modified layer, the response enhancement coefficients of the sensors to the four tested gases at 350 °C were drawn in Figure 12. All the response enhancement coefficients of the sensors with the modified layer to ethanol, acetone, and benzene were less than 1. With the increase of ZSM-5 modified layer, the response enhancement coefficients of the sensors with the modified layer to ethanol, acetone, and benzene decreased significantly and gradually. The variation of the response enhancement coefficients of the sensors with the modified layer to formaldehyde was a little perplexing. When the ZSM-5 layer was screen printed twice and 3 times, the response enhancement coefficients of the S(C/Z2) sensor and the S(C/Z3) sensor were approximately close to 1, which means the responses of the S(C/Z2) sensor and the S(C/Z3) sensor to 100 ppm formaldehyde had no difference with that of the S(C) sensor. Curiously, the response enhancement coefficients of the S(C/Z4) sensor and the S(C/Z5) sensor increased dramatically and reached 2.75 and 5.92 respectively. In other words, the response of the S(C/Z5) sensor with the ZSM-5 modified layer thickness of 19.5 μm to 100 ppm formaldehyde was 5.92 times that of the unmodified S(C) sensor at 350 °C. When the ZSM-5 modified layer thickness reached 31.8 μm, the response enhancement coefficient of the S(C/Z10) sensor closed to 1 again. The reason for the change in the response enhancement coefficients will be discussed below.

### 3.5. Stability

**Figure 13:**
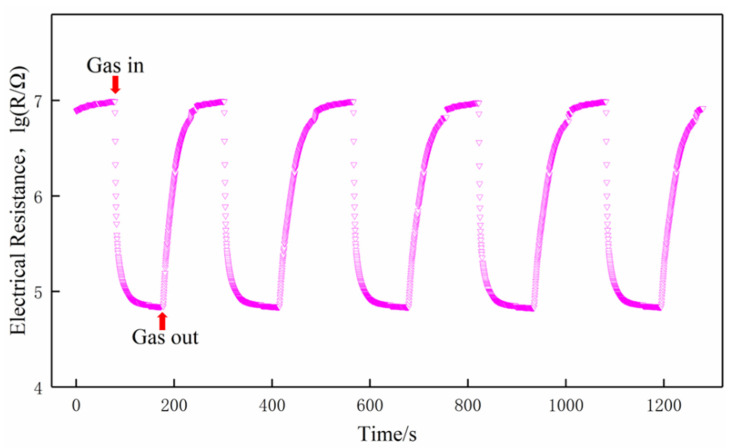
Cycle curve of the S(C/Z5) sensor on exposure to 100 ppm formaldehyde at 350 °C.

The sensors must have a good stability to meet the demand of practical applications [25]. In order to verify the stability and reliability of the test data, taking the S(C/Z5) gas sensor as an example, a cyclic test was performed on 100 ppm formaldehyde at 350 °C, and the transient response curve is depicted in Figure 13. It can be observed that the baseline resistance of the sensor decreased slightly between the second cycle and the first cycle. However, from the third cycle, the baseline resistance of the sensor was maintained at a stable value. Similar behaviors can be found in the work of other researchers [25,26]. This is attributed to the testing gas molecules remaining bonded to the sensitive materials and desorping incompletely although a recovery step is applied to the sensor [27]. Instead, under the same test conditions, the electrical resistance of the sensor to 100 ppm formaldehyde is quite stable in each measuring cycle. The result indicated that time could not influence the response regularity of the gas sensor, and the S(C/Z5) sensor had good stability.

## 4. Discussion on Gas Sensitive Mechanism

It is well known that zeolite plays a sieving and catalytic role when it interacts with gas molecules. The sieving effect is closely related to the dynamics diameters of gas molecules and the pore size in zeolite [28]. The gas molecular dynamics diameters of ethanol, acetone, benzene, and formaldehyde were 0.47 nm, 0.49 nm, 0.65 nm, and 0.24 nm, respectively [29]. Among the VOCs tested in the experiment, formaldehyde had the smallest gas molecular dynamics diameter, then the fastest diffusion speed in the ZSM-5 modified layer, and it was easier to reach the sensitive film to react with nano-SnO_2_. In addition, there were a large number of [AlO_2_] negative charges in ZSM-5, which had strong adsorption on polar gas molecules [30]. Benzene is a kind of nonpolar gas [31], which was difficult to be adsorbed by the ZSM-5 when it passes through the ZSM-5 modified layer. Meanwhile, benzene had the largest molecular dynamics diameter in this experiment, so it was difficult to pass through the ZSM-5 modified layer and reach the nano-SnO_2_ film [21]. Therefore, with the increase of the thickness of the ZSM-5 modified layer, the responses of the sensors decreased gradually.

Ethanol, acetone, and formaldehyde are polar gas molecules, and formaldehyde has the highest polarity [32]. The increase of the number of polar molecules adsorbed in the ZSM-5 modified layer [33] makes these gas molecules play a certain enrichment effect in the modified layer, which leads to the improvement of the responses of the sensors. However, besides the adsorption effect, ZSM-5 also has a large number of acidic sites, where the reaction of the tested gases with oxygen will be greatly catalyzed [34]. It was shown that ethanol is oxidized to ethylene and water (Equation (1)) with the catalysis of ZSM-5 at 300–400 °C [35]. The ethylene produced by the reaction will escape from the surface of the modified layer because ethylene is a kind of non-polar gas. Then the responses of the sensors with the ZSM-5 layer to ethanol decrease greatly. As can be seen from Figure 12, when the thickness of the modified layer was 31.8 μm, the response of the S(C/Z10) sensor to 100 ppm ethanol was only 11% of that of the S(C) sensor without the modified layer at 350 °C.

(1) $$CH_{3}CH_{2}OH+O_{\left(zeolite\right)}=C_{2}H_{4}+H_{2}O\;$$

(2) $$HCHO+2O_{\left(zeolite\right)}=CO_{2}+H_{2}O\;$$

(3) $$CH_{3}COCH_{3}+8O_{\left(zeolite\right)}=3CO_{2}+3H_{2}O\;$$

Equations (2) and (3) give the classical reaction equations of oxygen in ZSM-5 with acetone and formaldehyde gas molecules [21]. It is clear that the same concentration of formaldehyde produces less water than acetone, whereas acetone produces three times more water vapor than in the case of formaldehyde. The extra water vapor increases the humidity near the surface of the sensing materials and is detrimental to the sensor response [36]. This is why the responses of the sensors with the modified layer to acetone decrease.

**Figure 14:**
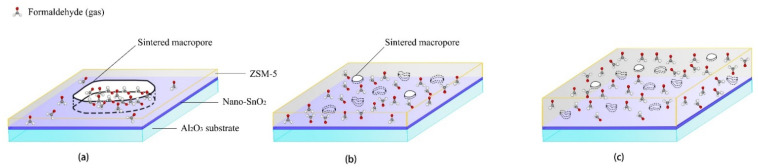
Schematic diagram showing the mechanism of formaldehyde sensing using the nano-SnO_2_ sensors with (**a**) thin and loose ZSM-5 layer, (**b**) medium and dense ZSM-5 layer, and (**c**) thick and dense ZSM-5 layer screen printed on their surfaces.

The thickness of the ZSM-5 modified layer is another important factor affecting the sensors performance. As mentioned above, only when the modified layer is repeatedly screen printed for 4 times and 5 times, the responses of the sensors with the ZSM-5 layer to 100 ppm formaldehyde will be greatly improved at 350 °C. When the ZSM-5 layer was screen printed only 2 and 3 times, the surface morphology results show that there were a large number of sintering macropores with the size of about 10 μm in the modified layer. Here the ZSM-5 modified layer is a kind of non-continuous membrane, and the size of the sintered macropores was about 10,000 times of that of the micropores in the ZSM-5 zeolite. Formaldehyde gas molecules can easily reach the surface of the nano-SnO_2_ sensitive film through the sintered macropores and react with the nano-SnO_2_. Now the sintering macropores play a major role in the ZSM-5 modified layer, which explains why the responses of the S(C/Z2) sensor and the S(C/Z3) sensor to formaldehyde were basically the same as that of the S(C) sensor (Figure 14a).

Although the thickness of the ZSM-5 layer was only increased by 1 μm and 3 μm compared with the S(C/Z3) sensor when the modified layer was screen printed 4 and 5 times, the density of the layers was significantly improved, and the size of the sintered macropores was also reduced to 2–3 μm. The sintering macropores could no longer penetrate the whole modified layer (Figure 14b). It is difficult for formaldehyde gas molecules to directly reach the nano-SnO_2_ sensitive layer through the sintered macropores. Then the micropores in ZSM-5 zeolite play a dominant role in molecular sieving, gases enrichment, and catalysis of the reactions [18,19,20,21,22,37]. It can be seen that the effect of the ZSM-5 modified layer was closely related to the integrity of the film. For screen printing technology, due to volatilization of printing oil, a single printing process can only obtain a non-continuous film [23,24], but multiple printing can offset the effect of sintering macropores through pore filling and paste extruding. The integral screen printed ZSM-5 layer without through holes could work as a continuous ZSM-5 film.

When the film thickness was further increased, the distance of formaldehyde gas molecules diffusion to the nano-SnO_2_ sensitive film increased. With the increase of diffusion distance, formaldehyde molecules in the ZSM-5 modified layer present a gradient distribution (Figure 14c). The concentration of formaldehyde in the upper part of the modified layer was quite high, whereas the concentration of formaldehyde was greatly reduced at the interface between the ZSM-5 layer and the nano-SnO_2_ film, which explains why the response of the S(C/Z10) sensor to formaldehyde decreased sharply when the ZSM-5 layer was very dense and the thickness of the ZSM-5 layer was 31.8 μm.

Table 3 summarizes the selectivity of various formaldehyde gas sensors. It is seen that the nano-SnO_2_ gas sensors with the ZSM-5 modified layers reported in this work exhibited excellent formaldehyde selectivity to ethanol, acetone, and benzene. It is worth noting that the sensors in Reference [22] and [37] are very similar to those in this work both in the materials and the structure of the sensors [22,37]. The formaldehyde selectivity to ethanol and acetone of the sensor in this work are 3.6 and 13.8 times to the SnO_2_/ZSM-5 composite nanofibers synthesized via an electrospinning technique, respectively [37]. Particularly, the formaldehyde selectivity to ethanol and acetone of the SnO_2_ sensor with the ZSM-5 coated layer are only 5.03 and 13.69, respectively [22], which are less than those in this work. The formaldehyde selectivity improvement of the nano-SnO_2_ gas sensor with the screen-printed layer is due to improvement of the thickness and density of the ZSM-5 modified layer. Simultaneously, considering the low cost of the screen printing technique, the sensors developed in this paper has a broad application prospect in formaldehyde detecting.

## 5. Conclusions

In this paper, nano-SnO_2_ was prepared by thermal decomposition. The SnO_2_ thick film sensors with commercial ZSM-5 modified layer were prepared by the screen printing technique. The results showed that when the surface of the nano-SnO_2_ sensitive film was screen printed 5 times with a mass ratio of 1:5 to the ZSM-5 zeolite and the printing oil, a dense ZSM-5 layer with the thickness of 19.5 μm was obtained. The response of the sensor with the 19.5 μm ZSM-5 layer to 100 ppm formaldehyde was 5.92 times higher than that of the unmodified sensor at 350 °C. The results show that the nano-SnO_2_ gas sensor with the ZSM-5 modified layer had strong selectivity for formaldehyde. The decrease of the sensor responses to benzene, ethanol, and acetone was closely related to the adsorption, sieving and catalysis of the ZSM-5 zeolite layer. The results also revealed that the integrity of the ZSM-5 modified membrane plays an important role in improving the selectivity of the sensors to formaldehyde. When the ZSM-5 modified layer was non-continuous, gas molecules directly pass through the layer and react with the sensitive materials through the sintering macropores, thus ignoring the role of micropores in ZSM-5. For the preparation technique of screen printing, multiple printing can improve the size and distribution of the sintering macropores in the film, and gradually transition from a non-continuous film to a continuous film. If the modified film is too thick, it will affect the diffusion process of gas molecules and reduce the selectivity of the sensors.

## Figures and Tables

**Table 1:** The structure of the gas sensors.

Sample Number	Substrate Layer	Modified Layer
S(C)	nano-SnO_2_	None
S(C/Z2)	nano-SnO_2_	2 layers of ZSM-5
S(C/Z3)	nano-SnO_2_	3 layers of ZSM-5
S(C/Z4)	nano-SnO_2_	4 layers of ZSM-5
S(C/Z5)	nano-SnO_2_	5 layers of ZSM-5
S(C/Z10)	nano-SnO_2_	10 layers of ZSM-5

**Table 2:** The ZSM-5 film thickness corresponding to different screen printing.

Sample Number	Modified Layer	ZSM-5 Film Thickness/μm
S(C)	None	-
S(C/Z2)	2 layers of ZSM-5	12.6
S(C/Z3)	3 layers of ZSM-5	16.5
S(C/Z4)	4 layers of ZSM-5	17.5
S(C/Z5)	5 layers of ZSM-5	19.5
S(C/Z10)	10 layers of ZSM-5	31.8

**Table 3:** Selectivity comparison of formaldehyde gas sensors.

Type	Materials	Selectivity of Formaldehyde (S_formaldehyde_/S_i_)				Ref.
		Ethanol	Acetone	Benzene	H_2_	
MOS + Zeolite	SnO_2_+ ZSM-5	15.59	21.09	34.6	-	This work
Metal + MOS	Zn-NiO	-	-	-	14.8	[6]
MOS + Zeolite	SnO_2_+ ZSM-5	5.03	13.69	-	-	[22]
MOS + Zeolite	SnO_2_+ ZSM-5	4.3	1.53			[37]
ZIF sensor	ZIF-8	-	6.25	-	-	[38]
	Zn(NA)	3.66	3.3	-	-	
	Zn(INA)	-	2.4	-	-	
ZIF coated-sensors	ZnO@ZIF-8	4.0	6.5	-	-	[7]

## Data Availability

The data presented in this study are available on request from the corresponding author. The data are not publicly available due to privacy.
